# Supplementary material for: Comprehensive CRISPR/Cas9-based mutagenesis identifies single-amino acid substitutions that abrogate SPEN function in X inactivation
Source: Nat Commun. 2026 Apr 6;17:4898. doi: 10.1038/s41467-026-71400-4 (PMC13230580; doi:10.1038/s41467-026-71400-4)
Supplement: Supplementary file 1 — Supplementary Information [file 41467_2026_71400_MOESM1_ESM.pdf]

SUPPLEMENTARY TABLES

Supplementary Table 1: Reagents and material used in this study. Listed are product name, product number, and manufacturer.

Product	Manufacturer	Prod. Nr.
β-mercaptoethanol (≥99.0%)	Sigma-Aldrich	M6250-250ML
4% Paraformaldehyde (PFA) in PBS	Santa Cruz Biotechnology	Sc281692
6-Thioguanine (6TG)	Sigma-Aldrich	A4660-2.5 (CAS: 205-8272)
Alt-R Cas9 Electroporation enhancer (10 nmol)	Integrated DNA Technology (IDT)	1075916
Alt-R HDR Enhancer V2	Integrated DNA Technology (IDT)	10007910
B27 supplement	Thermo Fisher Scientific	12587010
BsteII-HF	New England Biolabs (NEB)	R3162
CHIR99021	Axon Medchem	252917-06-9
Carboxylate-Modified Magnetic Particles	GE Life Sciences	GE65152105050250, GE45152105050250
CUT&RUN Assay Kit	Cell SignalingTechnology	86652
CUT&RUN H3K27ac ab	Cell Signaling Technology	8173
DMEM, high glucose (Gibco)	Thermo Fisher Scientific	41965039
DMEM/F12	Thermo Fisher Scientific	21041025
Doxycycline hyclate, ≥ 98% (HPLC)	Sigma-Aldrich	D9891-1G
Dynabeads protein G	Thermo Fisher Scientific	10004D
Fetal Bovine Serum	biowest	S1810-500 (Lot: S16927S1810)
Geltrex™ LDEV-Free, hESC-Qualified,	Thermo Fisher Scientific	A1413302
L-Glutamine	Thermo Fisher Scientific	25030-024
Human Fibronectin	Corning	356008
Hoechst 33342, trihydrochloride trihydrate, 10 mg/mL	Thermo Fisher Scientific	H3570
Hybrisol VII	MP Biomedicals	RIST1390
Knockout Serum Replacement	Thermo Fisher Scientific	10828010
Illumina Stranded mRNA Prep, Ligation	Illumina	20040532/4
Lipofectamine 3000 Transfection Reagent (Invitrogen)	Thermo Fisher Scientific	L3000008
MEM NEAA (Gibco)	Thermo Fisher Scientific	11140-035
Micrococcal nuclease	Cell signaling	10011S
N-2 supplement	Thermo Fisher Scientific	17502048
NDiff 227	Takara Bio	Y40002
NEBNext Ultra II DNA Library Prep Kit for Illumina	New England Biolabs (NEB)	7645
Neurobasal	Thermo Fisher Scientific	12348017
Nextera XT DNA Library Prep Kit	Illumina	FC-131-1024
NheI-HF	New England Biolabs (NEB)	R3131
NotI-HF	New England Biolabs (NEB)	R3189
PBS, pH 7.4 (Gibco)	Thermo Fisher Scientific	10010015
PD0325901	Axon Medchem	391210-10-9
Puromycin	Sigma-Aldrich	P8833-25MG
QIAquick Gel Extraction Kit	QIAGEN	28704
Recombinant human/mouse/rat Activin A PLUS protein	Qkine	QK005
Recombinant human FGF-2 (bFGF)	Qkine	QK025
Sodium pyruvate (Gibco)	Thermo Fisher Scientific	11360-039
T4 DNA Ligase	Thermo Fisher Scientific	EL0011
Tri-Methyl-Histone H3 (Lys27) (C36B11) Rabbit Monoclonal Antibody	Cell Signaling	9733
Triton X-100 (BioChemica)	ITW Reagents Division	A1388
TWEEN 20	Sigma-Aldrich	P1379-1L
VECTASHIELD Antifade Mounting Medium (with DAPI)	Vector Laboratories, Inc.	H-1200
XmaI	New England Biolabs (NEB)	R0180
Zymo ChIP DNA Clean Concentrator kit	Zymo Research	D5205
