# Supplementary material for: Comprehensive CRISPR/Cas9-based mutagenesis identifies single-amino acid substitutions that abrogate SPEN function in X inactivation
Source: Nat Commun. 2026 Apr 6;17:4898. doi: 10.1038/s41467-026-71400-4 (PMC13230580; doi:10.1038/s41467-026-71400-4)
Supplement: Supplementary file 4 — Description of Additional Supplementary Files [file 41467_2026_71400_MOESM4_ESM.docx]

**Description of Additional Supplementary Files**

Supplementary Data 1

Description: **sgRNA sequences used for base-editing screens on *Hprt*, *Msh2*, and *Spen*.** Listed are sgRNA identifiers used in this study, spacer sequence, the protospacer adjacent motif (PAM), the targeted exon, strand orientation, and genomic coordinates of each PAM site.

Supplementary Data 2

Description: **Long-range PCR primers used for the generation of amplicon-based libraries in the base-editing screens on *Hprt*, *Msh2*, and *Spen*.** Listed are PCR primer names used in this study, their 5’-3’ sequence, and the amplicon length of the given primer pair.

Supplementary Data 3

Description: **Phosphorylation site identification and quantification in enriched samples.** Listed are identified phosphopeptides detected by mass spectrometry, including protein and gene names, UniProt protein IDs, peptide sequences, and modified residue indices. Localization probabilities indicate confidence of phosphorylation site assignment. Localization probabilities and signal intensities are shown for enriched samples for wild-type cells with (Dox) and without (no Dox) doxycycline-induced Xist expression
